# Simplified urine-based method to detect rifampin underexposure in adults with tuberculosis: a prospective diagnostic accuracy study

**DOI:** 10.1128/aac.00932-23

**Published:** 2023-10-25

**Authors:** Yingda L. Xie, Nisha Modi, Deborah Handler, Sijia Yu, Prakruti Rao, Leonid Kagan, Kristen Petros de Guex, Robert Reiss, Anna Siemiątkowska, Anshika Narang, Navaneeth Narayanan, Jasie Hearn, Amanda Khalil, Patricia Woods, Laura Young, Alfred Lardizabal, Selvakumar Subbian, Charles A. Peloquin, Christopher Vinnard, Tania A. Thomas, Scott K. Heysell

**Affiliations:** 1 Department of Medicine, Rutgers New Jersey Medical School, Newark, New Jersey, USA; 2 Department of Pharmaceutics and Center of Excellence for Pharmaceutical Translational Research and Education, Ernest Mario School of Pharmacy, Rutgers, State University of New Jersey, Piscataway, New Jersey, USA; 3 Division of Infectious Diseases and International Health, University of Virginia, Charlottesville, USA; 4 Department of Physical Pharmacy and Pharmacokinetics, Poznan University of Medical Sciences, Poznań, Poland; 5 Virginia Department of Health, Richmond, USA; 6 New Jersey Department of Health, Trenton, USA; 7 College of Pharmacology, University of Florida, Gainesville, USA; 8 Rutgers New Jersey Medical School, Newark, USA; Bill & Melinda Gates Medical Research Institute, Cambridge, Massachusetts, USA

**Keywords:** rifampin, tuberculosis, pharmacokinetics, spectrophotometry, colorimetry, therapeutic drug monitoring

## Abstract

Variable pharmacokinetics of rifampin in tuberculosis (TB) treatment can lead to poor outcomes. Urine spectrophotometry is simpler and more accessible than recommended serum-based drug monitoring, but its optimal efficacy in predicting serum rifampin underexposure in adults with TB remains uncertain. Adult TB patients in New Jersey and Virginia receiving rifampin-containing regimens were enrolled. Serum and urine samples were collected over 24 h. Rifampin serum concentrations were measured using validated liquid chromatography–tandem mass spectrometry, and total exposure (area under the concentration–time curve) over 24 h (AUC_0–24_) was determined through noncompartmental analysis. The Sunahara method was used to extract total rifamycins, and rifampin urine excretion was measured by spectrophotometry. An analysis of 58 eligible participants, including 15 (26%) with type 2 diabetes mellitus, demonstrated that urine spectrophotometry accurately identified subtarget rifampin AUC_0–24_ at 0–4, 0–8, and 0–24 h. The area under the receiver operator characteristic curve (AUC ROC) values were 0.80 (95% CI 0.67–0.90), 0.84 (95% CI 0.72–0.94), and 0.83 (95% CI 0.72–0.93), respectively. These values were comparable to the AUC ROC of 2 h serum concentrations commonly used for therapeutic monitoring (0.82 [95% CI 0.71–0.92], *P* = 0.6). Diabetes status did not significantly affect the AUC ROCs for urine in predicting subtarget rifampin serum exposure (*P* = 0.67–0.92). Spectrophotometric measurement of urine rifampin excretion within the first 4 or 8 h after dosing is a simple and cost-effective test that accurately predicts rifampin underexposure. This test provides critical information for optimizing tuberculosis treatment outcomes by facilitating appropriate dose adjustments.

## INTRODUCTION

Rifampin is a key sterilizing drug in standard tuberculosis (TB) regimens that enables cure of most drug-susceptible TB within 6 mo. However, there is extensive pharmacokinetic (PK) variability in absorption and metabolism of anti-TB drugs including rifampin, with low drug concentrations associated with poor treatment outcomes including treatment failure, relapse, and acquired resistance (1, 2). To guide effective treatment in situations with poor response, drug toxicity, or suspected underexposure, therapeutic drug monitoring is a critical component of the American Thoracic Society/Centers for Disease Control and Prevention/Infectious Diseases Society of America (3) and World Health Organization TB treatment guidelines (4).

Therapeutic drug monitoring remains inaccessible in many high-burden, resource-constrained settings due to the need for expensive and specialized equipment, such as high-performance liquid or gas chromatography, or mass spectrometry. The requirement for shipping, cold chain, and sample processing further hampers the implementation of personalized dosing based on one’s own pharmacokinetics, leading to missed opportunities to address inadequate drug exposures and improve treatment outcomes. A simple, inexpensive test that could be integrated into a point-of-care diagnostic or even performed in a peripheral laboratory could provide same-day or next-day results to inform critical dose adjustments early in treatment. Urine spectrophotometry is noninvasive, has low cost, has a stable reagent, and has been implemented for qualitative detection of isoniazid in the context of adherence monitoring (“Arkansas method”) (5, 6). In previous pilot studies, we found that urine spectrophotometry accurately identified adult TB patients co-infected with HIV with low rifampin AUC_0–24_ exposures (4). However, to date, there has been limited systematic evaluation of urine spectrophotometry across different post-dose time intervals to predict serum rifampin underexposure in a larger prospective adult TB cohort.

## MATERIALS AND METHODS

### Settings and participants

The study enrolled adults undergoing treatment for active TB with a first-line rifampin-containing regimen during an intensive (rifampin, isoniazid, and pyrazinamide±ethambutol) or continuation (rifampin+isoniazid) phase of anti-TB therapy at recommended doses (3). Participants were recruited by chart review and referrals from New Jersey (NJ) (Rutgers Global TB Institute’s Lattimore Practice serving Essex and Union Counties as well as Middlesex, Hudson, and Patterson county clinics covering approximately 50% of the NJ active TB population) and Virginia (University of Virginia and surrounding Virginia Department of Health clinics). Sixty participants were targeted for recruitment to adjust for prespecified covariables (sex, diabetes). Both sites had capacity for therapeutic drug monitoring by serum analyses, and thus, the procedures were in place for processing, handling, storing, and shipping patient samples for purposes of drug concentration assays. Human subject approval was obtained through Rutgers Health Sciences IRB Pro2018001857 and University of Virginia Health Sciences IRB HSR #20944.

### Serum collection and drug quantification

Oral doses of the anti-TB drugs were administered under direct observation on the morning of the pharmacokinetics assessment visit. A baseline blood sample was drawn prior to medication administration (time 0), then at 1, 2, 4, 6, and 8 h post-dose (7). Blood samples were processed on-site and frozen until shipment to the Infectious Disease Pharmacokinetics Laboratory at the University of Florida. Rifampin serum concentrations were quantified using validated liquid chromatography–tandem mass spectrometry procedures (8, 9) with the lower limit of quantification of 0.25 mg/L. Cmax was recorded as the highest of the measured rifampin concentrations during the dosing interval. Total serum exposure, defined as the area under the serum concentration time curve over 24 h (AUC_0–24_), was determined by noncompartmental analysis using Phoenix WinNonlin version 8.3 (Certara, Princeton, New Jersey, USA) (10, 11).

### Urine collection and spectrophotometric assays

Urine was collected at baseline (predose) and 0–4, 4–8, and 8–24 h (post-dose). Separate graded receptacles were used for each urine collection interval. At 0–4 and 4–8 h, aliquots were drawn from the collection receptacle, and the total urine volume at each timepoint was noted. Three different urine collection periods were examined: 0–4, 0–8, and 0–24 h. Proportional volumes of urine collected at 0–4, 4–8, and 8–24 h post-dose were pooled to determine concentrations at 0–8 and 0–24 h to reflect potential collection times in a clinical setting.

The Sunahara method was followed to extract total rifamycins, including rifampin and its metabolites, in urine (12). For every 500 µL of urine, 250 µL of 100 mM phosphate buffer (pH 7, Sigma Aldrich) and 500 µL of isoamyl alcohol (Sigma Aldrich) were added and run in triplicate per timepoint. The samples were vortexed for 20 s and centrifuged at maximum speed (10,000+ rpm) for 5 min at room temperature. Next, 100 µL of the aqueous phase (upper layer) was removed and added to a 96-well plate, and the optical density was measured at 475 nm using a spectrophotometer (BioTek, Synergy). A calibration curve was generated to derive the method quantification limit to quantify extracted rifampin from urine.

### Data analysis

Demographic and clinical characteristics and overall distribution of measured serum concentrations and urine drug amounts were evaluated using descriptive statistics. Individual rifampin concentration-versus-time profiles were plotted for each participant, and the corresponding rifampin Cmax for each individual was identified. Noncompartmental analysis was performed to calculate rifampin serum AUC_0–24_ for each participant that completed the study visit. For our primary analysis, we evaluated receiver operating characteristic (ROC) curves for the urine spectrophotometric assay corresponding to an AUC_0–24_ target of 35.4 mg·h/L, representing a contemporary consensus drug exposure target (13). A prospective study of adults with drug-susceptible pulmonary TB from South Africa found a rifampin serum AUC_0–24_ target of ≤13 mg·h/L, predictive of poor treatment outcomes (14), and thus, ROC curves were also generated for urine excretion values and this target in supplementary analyses. Furthermore, conventional serum 2 h concentrations (C2h), as estimates for a minimum Cmax target of 8 mg/L, were used as a comparator to urine excretion values, as a serum 2 h concentration has been used operationally in outpatient clinical or programmatic settings for rifampin dose adjustment in the absence of more extensive sampling needed for AUC_0–24_ calculation (15). The 95% confidence interval (CI) for the area under the ROC curves was calculated using 2,000 stratified bootstrap replicates. A minimum sensitivity threshold of 80% and 90%, as well as the maximum Youden *J* statistic, was used to define the optimal cutoff for the urine spectrophotometry, and sensitivity and specificity for prediction of subtarget concentrations were calculated using this cutoff. *P*-values were calculated based on a null hypothesis that AUC ROC = 0.5.

An adjusted ROC analyses was performed to adjust for key biologic variables, including the presence of type 2 diabetes mellitus (“diabetes”) and creatinine clearance. Diabetes status was defined in this study as a measured HbA1c ≥ 6.5% or an established diagnosis of diabetes on treatment, regardless of HbA1c. Data were analyzed in R software (version 3.6.1, http://r-project.org), with ROC analysis performed using the pROC package (16).

## RESULTS

### Clinical and demographic characteristics

Sixty adults with TB were enrolled of whom 59 were taking rifampin, and 58 completed all procedures. Baseline characteristics for these 58 participants are shown in Table 1. The mean age was 43.5 y, 38% (22/58) were female, 26% (15/58) had diabetes, and three individuals had a CrCl <60 mL/min. The majority of participants exceeded 14 d of rifampin treatment (96.6%; 56/58) at the time of PK sampling and were taking rifampin at a daily dose of 600 mg (86.2%; 50/56).

**TABLE 1:** Participant baseline characteristics

	All participants (*N* = 58)
Site, *n* (%)	
Virginia	14 (24.1%)
NJ	44 (75.9%)
Age (mean ± SD)	43.5 ± 15.2
Sex, *n* (%)	
Male	36 (62.1%)
Female	22 (37.9%)
Ethnicity, *n* (%)	
Hispanic	29 (50.0%)
Non-Hispanic	29 (50.0%)
Race, *n* (%)	
Asian	11 (19.0%)
Black or AA	13 (22.4%)
Caucasian	28 (48.3%)
Other	6 (10.3%)
BMI (kg/m^2^) (mean ± SD)	25.0 ± 7.3
Diabetes, *n* (%)	15 (25.9%)
HIV, *n* (%)	1 (1.7%)
CrCl (mL/min) (median [IQR])	120.2 [52.8]
≥60	52 (89.7%)
<60	3 (5.2%)
Not available	3 (5.2%)
Regimen, *n* (%)	
Intensive phase	51 (87.9%)
Continuation phase	7 (12.1%)
Days on rifampin (median [IQR])	50.0 [25.8]
≥14 d	56 (96.6%)
< 14 d	2 (3.4%)
Rifampin dose, *n* (%)	
1,200 mg	1 (1.7%)
900 mg	1 (1.7%)
600 mg	50 (86.2%)
450 mg	3 (5.2%)
300 mg	3 (5.2%)

### Serum pharmacokinetics

**Fig 1:**
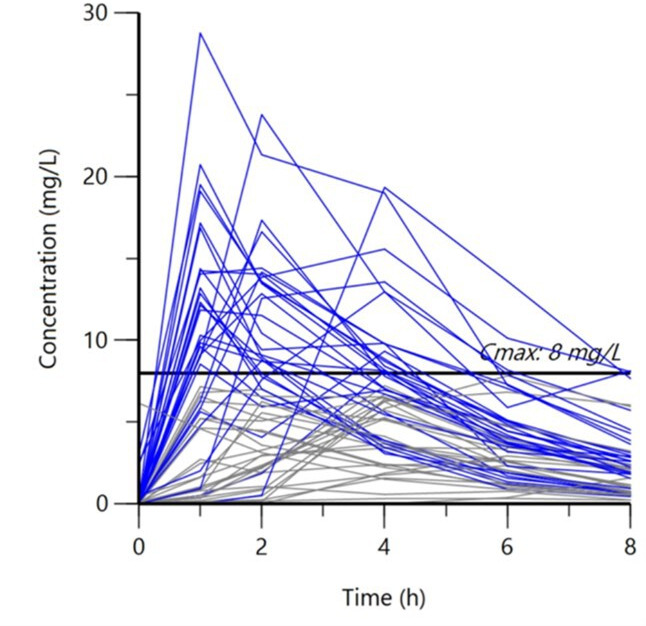
Individual serum rifampin concentration–time curves (mg/L) over the 8 h serum collection period. Time 0 designates time of the last rifampin dose. Blue lines designate participants who met or exceeded the Cmax 8 mg/L target conventionally used in the absence of AUC_0–24_ (total serum exposure) values; gray lines depict participants who were under the Cmax target.

Serum rifampin concentrations for the 58 individuals are shown in Fig. 1. Thirty (51.7%) participants reached the serum Cmax target of 8 mg/L while 36 (62.1%) participants reached the serum AUC_0–24_ target of 35.4 mg·h/L. The proportion of participants below the AUC_0–24_ target did not significantly differ between those with and without diabetes (diabetes 46.7% [7/15] vs nondiabetes 34.9% [15/43], *P* = 0.539 by Fisher’s exact test).

### Urine rifampin excretion by spectrophotometry

**Fig 2:**
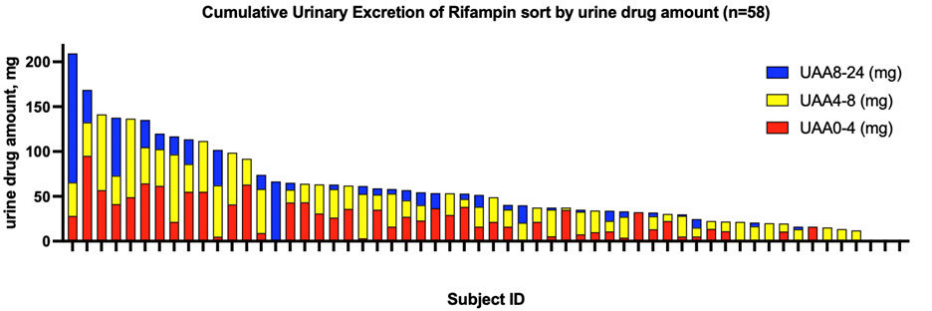
Cumulative urinary excretion of rifampin in milligrams over 24 h post-dose as measured by urine spectrophotometry. Each bar represents a participant (*n* = 58) in the study.

The calibration curve generated to quantify extracted rifampin from urine via the Sunahara method demonstrated a linear relationship (*r^2^* = 1) between rifampin concentration from 31.2 to 1,000 mg/L at the University of Virginia laboratory and between 7.8 and 1,000 mg/L at the Rutgers laboratory, due to different instrument detection limits. Urine from five participants was evaluated for assay reproducibility at both institutions, and the calculated rifampin concentration variation was under 10%. The distribution of urine excretion amounts by participant is shown in Fig. 2. The majority of participants excreted the greatest proportion of rifampin in the urine within the first 8 h. Three participants had urine rifampin excretion below the limit of quantification throughout the 24 h. These participants also had below target serum concentrations, with AUC_0–24_ values of 2.4, 21.4, and 31.9 mg·h/L. There was no association between urine excretion and number of days on rifampin treatment (0 > *R* > −0.029).

**Fig 3:**
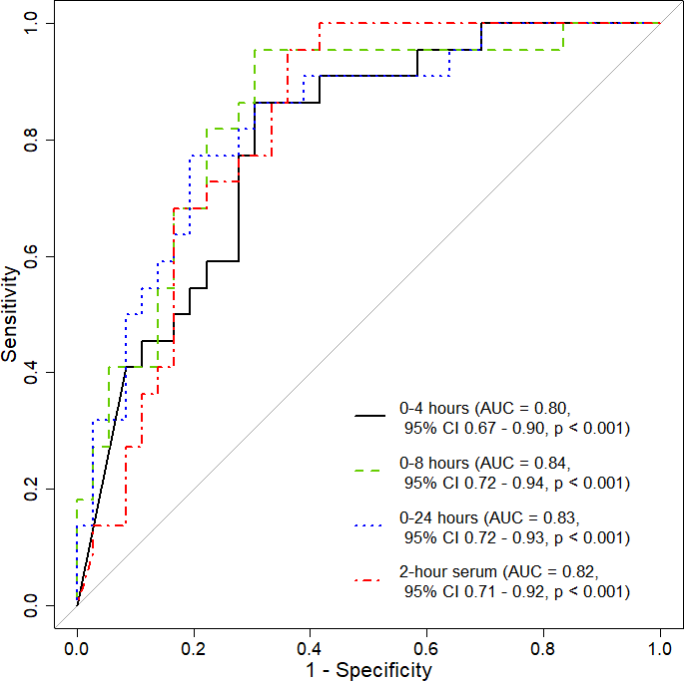
Receiver operator characteristic (ROC) curve for rifampin urinary dose excretion over 0–4, 0–8, and 0–24 h and 2 h serum prediction of total serum exposure in the dosing interval–area under the concentration time curve (AUC_0–24_) of less than 35.4 mg·h/L for the *n* = 58 participants.

For predicting those participants with serum rifampin below the minimum target AUC_0–24_ of 35.4 mg·h/L, the area under the ROC curve of urine rifampin excretion from 0 to 4 hours was 0.80 (95% CI 0.67–0.90, *P* < 0.01), 0–8 h 0.84 (95% CI 0.72–0.94, *P* < 0.01), and 0–24 h 0.83 (95% CI 0.72–0.93, *P* < 0.01) (Fig. 3). There was no difference in performance between urine excretion at any time interval versus 2 h serum concentration (C2h) in predicting AUC_0–24_ of less than 35.4 mg·h/L (C2h vs urine, *P* = 0.60 for urine 0–4 h, 0.71 for urine 0–8 h, and 0.87 for urine 0–24 h; Table S1).

Only four participants had an AUC_0–24_ below the target of 13 mg·h/L. Urine excretion maintained an AUC ROC >0.8 in predicting serum AUC_0–24_ below the target of 13 mg·h/L, although serum 2 h concentration tended to be more predictive at this threshold with an AUC ROC of 0.94 (C2h vs urine *P* = 0.044 for urine 0–4 h, 0.090 for urine 0–8 h, and 0.098 for urine 0–24 h; Fig. S1).

### Adjusted analysis for creatinine clearance, diabetes, and sex

In a post hoc ROC analysis, we excluded the three participants with CrCl <60 mL/min due to the substantial discordance between their high serum AUC exposures versus lower urine drug concentrations (Fig. S2). Among the remaining 55 individuals with CrCl >60 mL/min, the AUC ROCs of urine 0–4, 0–8, and 0–24 were 0.81 (95% CI 0.69–0.91, *P* < 0.01), 0.86 (95% CI 0.73–0.95, *P* < 0.01), and 0.85 (95% 0.73–0.94, *P* < 0.01), respectively (Fig. S3).

Diabetes and sex had no significant impact on the AUC for ROC curve of urine at any interval or serum 2 h concentration for predicting AUC_0–24_ < 35.4 mg·h/L (Fig. S4 and S5). Urinalysis profiles associated with diabetes were considered with regard to potential impact on rifampin excretion and measurement. Among the 14 participants with diabetes versus 39 without diabetes with available urinalysis data, 35.7% (5/14) vs 23.1% (9/39) had trace to 2+ proteinuria, 57.1% (8/14) vs 0% (0/39) had glucosuria, and average pH was 5.9 and 5.8, respectively.

### Potential clinical impact of urine spectrophotometry

Given the wide therapeutic window and tolerability of rifamycins (17, 18), we examined a high sensitivity threshold for the urine excretion values in which capturing subtarget serum exposure and dose escalation is emphasized over preventing unnecessary dose increases. Table 2 shows the specificity for predicting subtarget AUC_0–24_ across each urine time interval and serum 2 h when setting the sensitivity threshold to 80% or 90% and at the maximum Youden index. At minimum sensitivity thresholds of 80% or 90%, specificity of urine spectrophotometry including at 0–4 and 0–8 intervals met or exceeded that of serum 2 h values. At the maximum Youden index, sensitivity exceeded 86% for urine at 0–4 and 0–8 h.

**TABLE 2:** Specificity for predicting subtarget AUC _0-24_ at various thresholds

	Prediction: serum AUC_0–24_ < 35.4 mg·h/L		
Threshold	Measure	Specificity (%) (95% CI) n/n^*a*^	Sensitivity (%) (95% CI) n/n^*a*^
At least 80% sensitivity^*b*^	0–4 h	0.69	0.82
	(*n* = 58)	(0.42, 0.86)	(0.41, 1.00)
		25/36	18/22
	0–8 h	0.78	0.82
	(*n* = 58)	(0.58, 0.92)	(0.32, 1.00)
		28/36	18/22
	0–24 h	0.72	0.82
	(*n* = 58)	(0.39, 0.92)	(0.41, 0.95)
		26/36	18/22
	Serum 2 h	0.67	0.82
	(*n* = 58)	(0.53, 0.89)	(0.55, 1.00)
	(comparator)	24/36	18/22
At least 90% sensitivity^*b*^	0–4 h	0.58	0.91
	(*n* = 58)	(0.25, 0.81)	(0.68, 1.00)
		21/36	20/22
	0–8 h	0.69	0.91
	(*n* = 58)	(0.14, 0.86)	(0.59, 1.00)
		25/36	20/22
	0–24 h	0.61	0.91
	(*n* = 58)	(0.22, 0.86)	(0.73, 1.00)
		22/36	20/22
	Serum 2 h	0.64	0.91
	(*n* = 58)	(0.47, 0.83)	(0.59, 1.00)
	(comparator)	23/36	20/22
Youden index (max)	0–4 h	0.69	0.86
	(*n* = 58)		
	0–8 h	0.69	0.95
	(*n* = 58)		
	0–24 h	0.81	0.77
	(*n* = 58)		
	Serum 2 h	0.64	0.95
	(*n* = 58)		
	(comparator)		

^*a*^ n/n for sensitivity refers to true positive/(true positive+false negatives) where “positive” refers to subtarget AUC_0–24._; n/n for specificity refers to true negatives/(true negatives+false positives).

^*b*^ True positives set at the lowest number required to meet the 0.8 and 0.9 sensitivity thresholds.

## DISCUSSION

Spectrophotometric measurement of urine rifampin excretion over 24 h, even in the first 4 and 8 h post-dose, accurately predicted relevant subtarget rifampin serum exposures with an AUC ROC of 0.8 or greater and was no different than that of a 2 h serum concentration for predicting a clinically relevant target of total serum exposure. In contrast to collection and measurement of serum concentrations, urine spectrophotometry is a low-cost method without the need for specialized expertise or equipment and imminent potential to be developed for point-of-care or near-point-of-care use.

While the impact of urine spectrophotometry would rely on an effective dose adjustment model based on urine excretion values, high-dose rifampin of up to 1,200 mg with AUC_0–24_ values averaging 308 mg·h/L have been tolerated with mild, grade 1–2 hepatotoxicity (17, 18). Excluding those with renal impairment (CrCl <60 mL/min), individuals who would have been falsely classified as subtarget at the 80% and 90% sensitivity thresholds had serum AUC_0–24_ ranging from 35 to 72 mg·h/L. This suggests that even in the setting of an unnecessary dose increase based on urine excretion values for patients with serum AUC_0–24_ near the minimum target, a broad window exists before dose increase would lead to a potentially toxic serum exposure for those patients. Thus, in most circumstances, a gain in sensitivity prompting correction of underexposure may outweigh the decrement in specificity and false positive calls. We observed a similar sensitivity and specificity tradeoff with the conventional serum 2 h level.

Ultimately, a decision to proceed with dose adjustment based on urinary excretion or even limited serum testing such as that performed with a 2 h serum values is weighted by multiple clinical factors. These factors include the presence of significant renal dysfunction that may preclude the use of a urine-based assay for the measurement of drug excretion, TB disease severity and the likelihood of poor outcomes without optimal pharmacokinetics, knowledge of *M. tuberculosis* quantitative susceptibility testing (higher minimum inhibitory concentrations within the conventionally susceptible range) (19), and resources for close clinical monitoring following a dose increase. Nevertheless, personalizing a dose closer to the point of care for critical drug classes such as the rifamycins may be all the more important as TB treatment shortening regimens are implemented outside the cohorts of their original controlled trials (20, 21).

One limitation of this study is the modest sample size to perform further subgroup analyses. Despite this limitation, we observed that urine spectrophotometry performed well in both patients with and without diabetes despite observed differences that were expected in those groups with regard to urinalysis profiles. We have recently demonstrated similar predictive performance for serum rifampin exposures at different urine collection intervals among a more malnourished cohort of children treated for TB in Tanzania (22). Future prospective interventional studies are needed to test dose adjustment algorithms based on urinary excretion and serum target attainment to determine the generalizability and clinical impact. Additionally, while urine spectrophotometry performed in this study bypasses the need for specialized chemistry labs, the need for pipetting and power requirements for centrifugation and spectrophotometry still require a basic laboratory facility. Work is ongoing to further streamline the processing steps and spectrophotometric readouts into a simplified and portable workflow operated by battery power and amenable to use at the point-of-care (23).

In conclusion, urine spectrophotometry can accurately diagnose rifampin serum pharmacokinetic underexposure. The noninvasive and low-cost characteristics of urine spectrophotometry make it a preferential option for use in TB endemic or programmatic settings that previously have not benefited from personalized dosing. Urine spectrophotometry could be scaled up to ensure adequate minimal drug exposure in all patients by continued used of fixed-dose combinations plus single additive rifampin tablets, thereby ameliorating one of the few correctable determinants of TB treatment outcomes.

## Data Availability

Study dataset is available in Supplementary materials (Supplemental file 2).

## SUPPLEMENTAL MATERIAL

Supplemental file 1 aac.00932-23-s0001.pdf Supplemental figures and tables.

Supplemental file 2 aac.00932-23-s0002.xlsx

ASM does not own the copyrights to Supplemental Material that may be linked to, or accessed through, an article. The authors have granted ASM a non-exclusive, world-wide license to publish the Supplemental Material files. Please contact the corresponding author directly for reuse.
