# Supplementary material for: Simplified urine-based method to detect rifampin underexposure in adults with tuberculosis: a prospective diagnostic accuracy study
Source: Antimicrob Agents Chemother. 2023 Oct 25;67(11):e00932-23. doi: 10.1128/aac.00932-23 (PMC10648923; doi:10.1128/aac.00932-23)

Supplemental Material

Table S1. Pairwise comparisons represented as p-values of AUC ROC for predicting $AUC_{0\text{-}24} < 35.4$ between urine 0-4, 0-8, 0-24, and serum 2-hour rifampin concentrations.

Unadjusted p-values	0-4 hours (n = 58)	0-8 hours (n = 58)	0-24 hours (n = 58)
0-8 hours (n = 58)	0.23		
0-24 hours (n = 58)	0.51	0.78	
Serum 2 hour (n = 58) (comparator)	0.60	0.71	0.87

Figure S1. Receiver Operator Characteristic (ROC) curve for rifampin urinary dose excretion over 0-4, 0-8, 0-24 hours and 2-hour serum prediction of calculated serum area under the concentration time curve (AUC_{0-24}) of less 13 mg·h/L

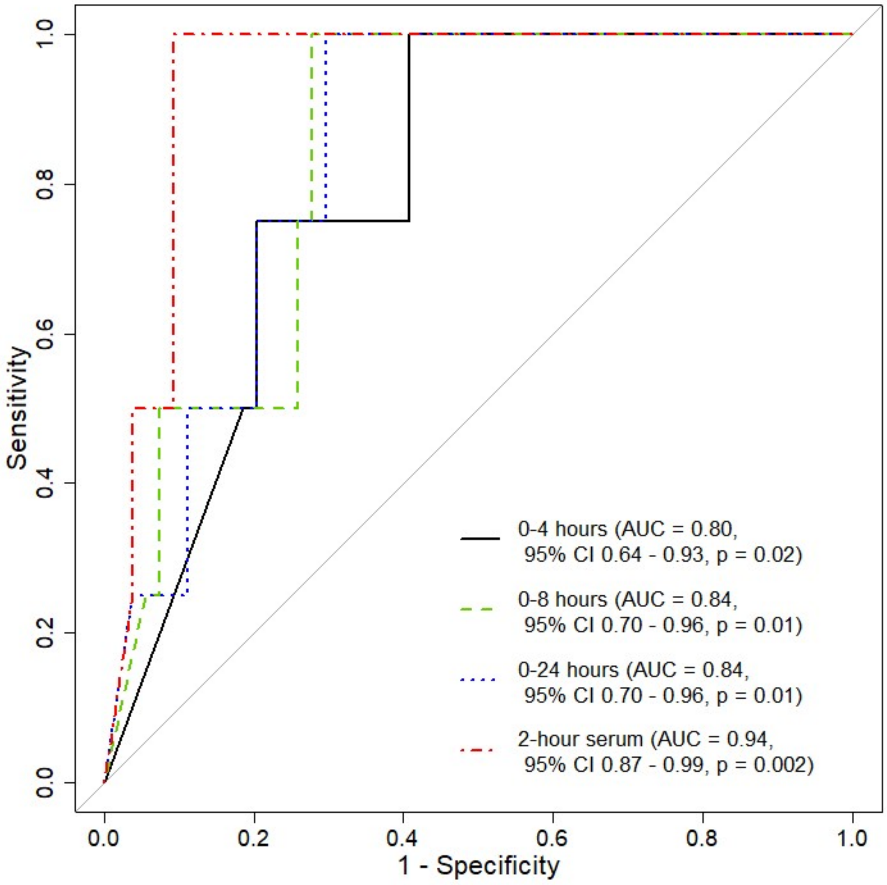

Figure S2. Correlation of serum vs urine values with (A) all participants in the analysis population, with individuals CrCl < 60 (participants # 149, 259, 309) demarcated by the blue label, and (B) exclusion of participants with CrCl < 60.

A.

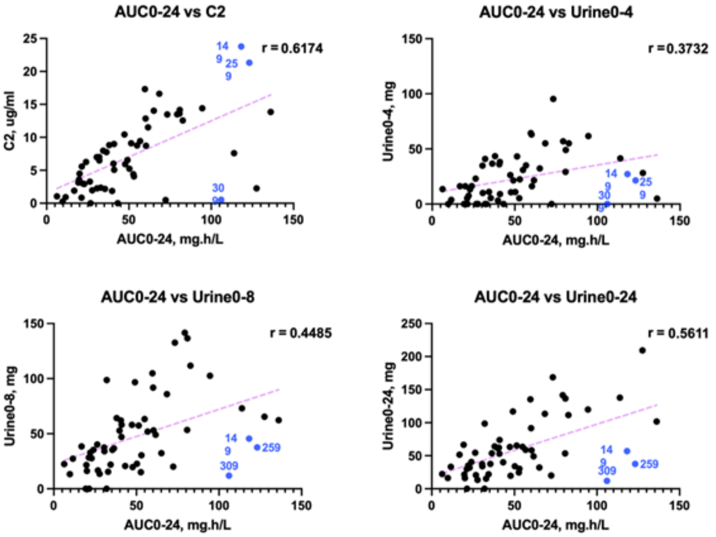

B.

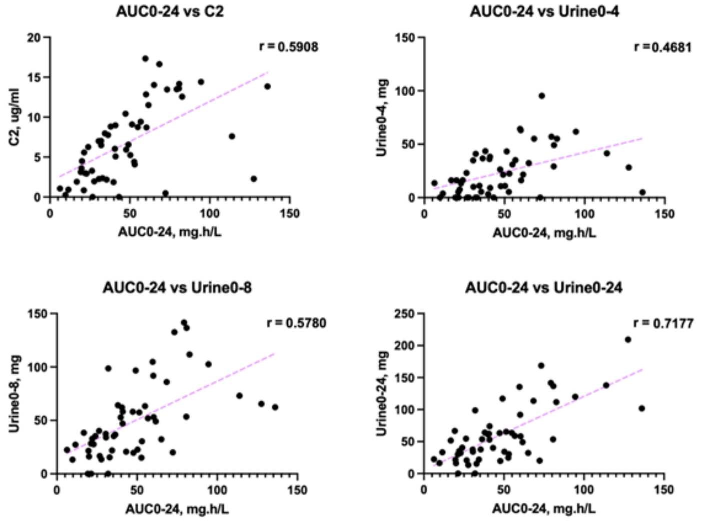

Figure S3. Receiver Operator Characteristic (ROC) curve for rifampin urinary dose excretion over 0-4, 0-8, 0-24 hours and 2-hour serum prediction of calculated serum area under the concentration time curve (AUC_{0-24}) of less than (A) 35.4 mg·h/L and (B) 13 mg·h/L after exclusion of 3 participants with CrCl<60 (n=55).

A.

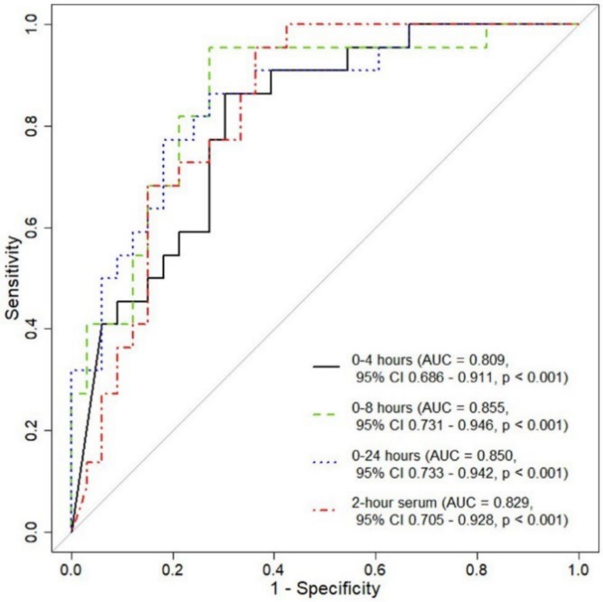

B.

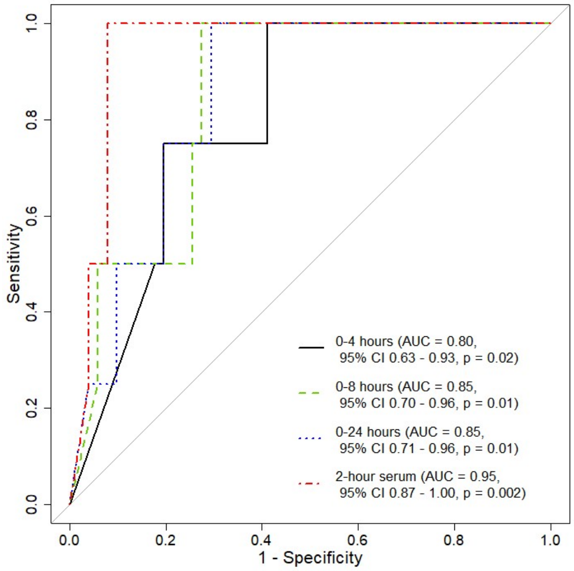

Figure S4. ROC for predicting AUC < 35.4 mg·h/L, stratified by diabetes status.

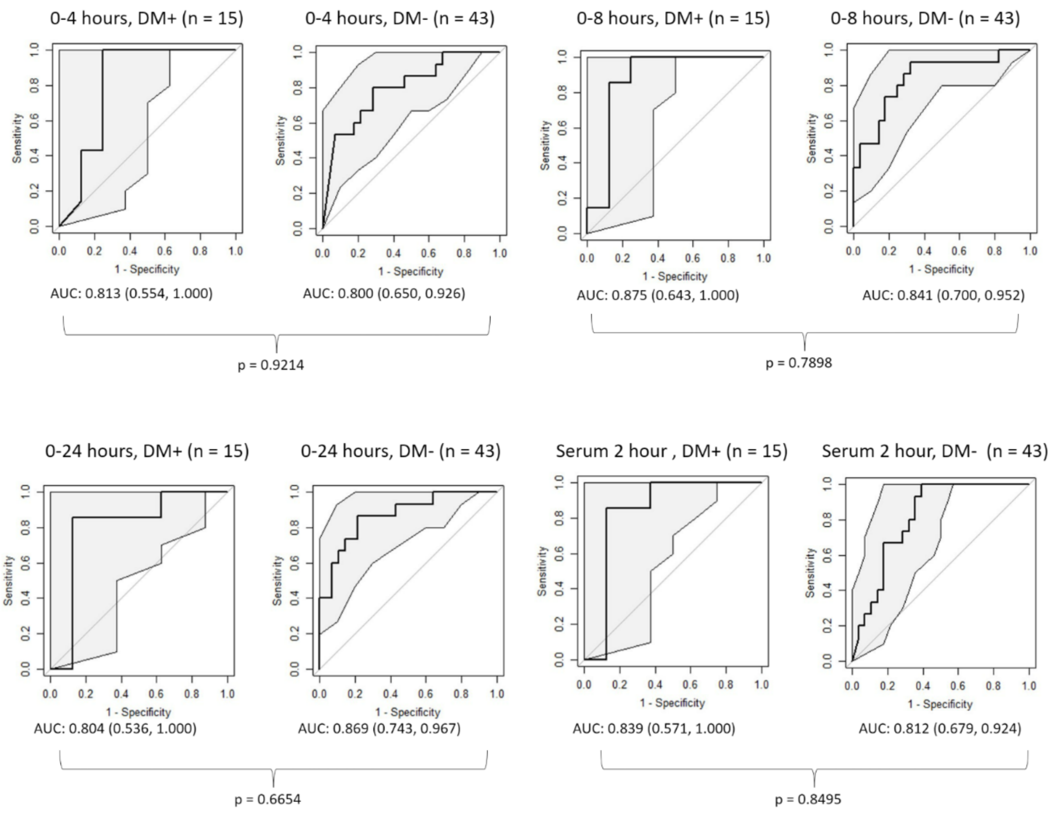

Figure S5. ROC for predicting AUC < 35.4 mg·h/L, stratified by sex.

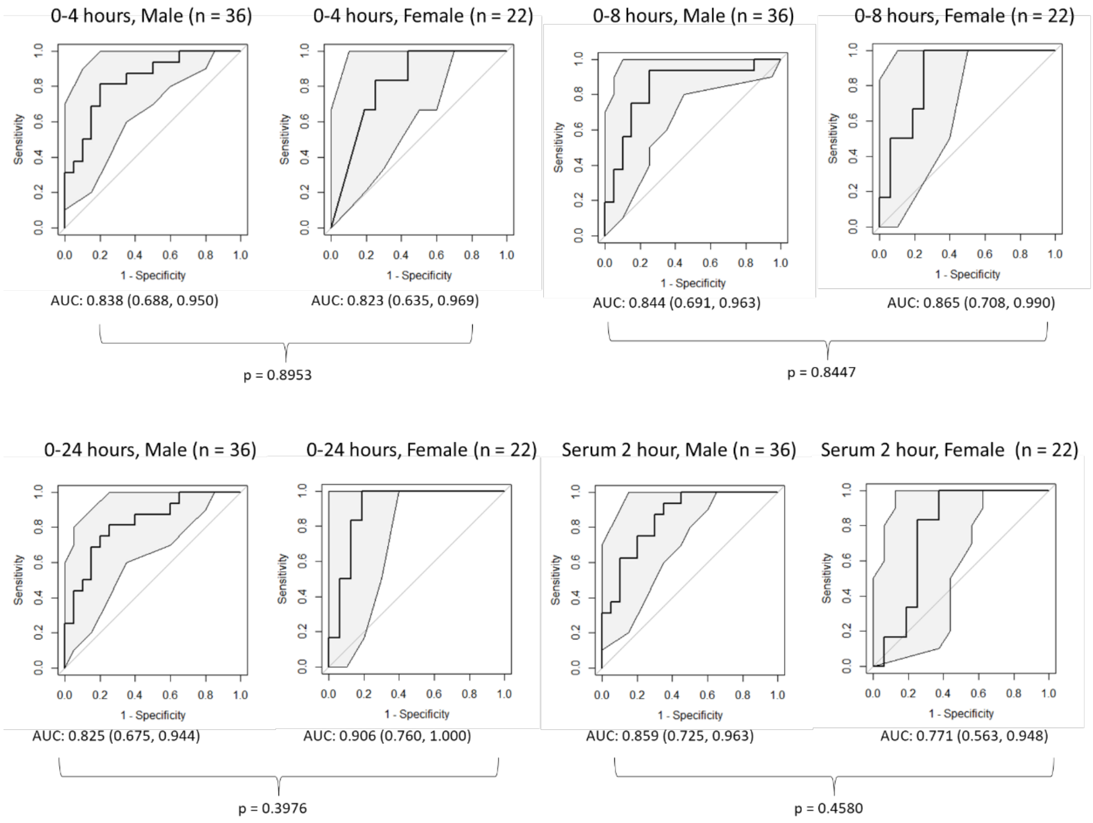

Supplement: Supplemental file 1 — Supplemental figures and tables. [file aac.00932-23-s0001.pdf]
